# Natural resistance to ascorbic acid induced oxidative stress is mainly mediated by catalase activity in human cancer cells and catalase-silencing sensitizes to oxidative stress

**DOI:** 10.1186/1472-6882-12-61

**Published:** 2012-05-02

**Authors:** Christoph Klingelhoeffer, Ulrike Kämmerer, Monika Koospal, Bettina Mühling, Manuela Schneider, Michaela Kapp, Alexander Kübler, Christoph-Thomas Germer, Christoph Otto

**Affiliations:** 1 Experimental Surgery, Department of Surgery, University of Würzburg Hospital, Oberdürrbacher Str. 6, D-97080, Würzburg, Germany; 2 Department of Obstetrics and Gynaecology, University of Würzburg Hospital, Josef-Schneider-Str. 4, D-97080, Würzburg, Germany; 3 Department of Oral and Maxillofacial Surgery, University of Würzburg Hospital, Pleicherwall 2, D-97070, Würzburg, Germany; 4 Department of Surgery, University of Würzburg Hospital, Oberdürrbacher Str. 6, D-97080, Würzburg, Germany

## Abstract

**Background:**

Ascorbic acid demonstrates a cytotoxic effect by generating hydrogen peroxide, a reactive oxygen species (ROS) involved in oxidative cell stress. A panel of eleven human cancer cell lines, glioblastoma and carcinoma, were exposed to serial dilutions of ascorbic acid (5-100 mmol/L). The purpose of this study was to analyse the impact of catalase, an important hydrogen peroxide-detoxifying enzyme, on the resistance of cancer cells to ascorbic acid mediated oxidative stress.

**Methods:**

Effective concentration (EC_50_) values, which indicate the concentration of ascorbic acid that reduced the number of viable cells by 50%, were detected with the crystal violet assay. The level of intracellular catalase protein and enzyme activity was determined. Expression of catalase was silenced by catalase-specific short hairpin RNA (sh-RNA) in BT-20 breast carcinoma cells. Oxidative cell stress induced apoptosis was measured by a caspase luminescent assay.

**Results:**

The tested human cancer cell lines demonstrated obvious differences in their resistance to ascorbic acid mediated oxidative cell stress. Forty-five percent of the cell lines had an EC_50_ > 20 mmol/L and fifty-five percent had an EC_50_ < 20 mmol/L. With an EC_50_ of 2.6–5.5 mmol/L, glioblastoma cells were the most susceptible cancer cell lines analysed in this study. A correlation between catalase activity and the susceptibility to ascorbic acid was observed. To study the possible protective role of catalase on the resistance of cancer cells to oxidative cell stress, the expression of catalase in the breast carcinoma cell line BT-20, which cells were highly resistant to the exposure to ascorbic acid (EC_50_: 94,9 mmol/L), was silenced with specific sh-RNA. The effect was that catalase-silenced BT-20 cells (BT-20 KD-CAT) became more susceptible to high concentrations of ascorbic acid (50 and 100 mmol/L).

**Conclusions:**

Fifty-five percent of the human cancer cell lines tested were unable to protect themselves against oxidative stress mediated by ascorbic acid induced hydrogen peroxide production. The antioxidative enzyme catalase is important to protect cancer cells against cytotoxic hydrogen peroxide. Silenced catalase expression increased the susceptibility of the formerly resistant cancer cell line BT-20 to oxidative stress.

## Background

Ascorbic acid (vitamin C), an essential nutrient for mammalian cells, acts as a cofactor of different enzymatic reactions, e.g. collagen synthesis. In addition, ascorbic acid has an important impact on oxidative stress caused by reactive oxygen species (ROS). Some of the most common ROS are superoxide anion, hydroxide radical and hydrogen peroxide [1]. The production of ROS is an inevitable outcome of aerobic respiration in mitochondria where oxygen acts as electron acceptor. Disturbances in aerobic respiration can lead to oxidative stress by the production of ROS, resulting in cellular senescence and apoptosis [2,3]. Antioxidant enzymes, part of the physiological defence mechanisms in mammalian cells against high concentrations of ROS, detoxify ROS into less toxic or inert molecules [4,5]. One prominent hydrogen peroxide-detoxifying enzyme is catalase.

Different studies showed a toxic effect of extracellular ascorbic acid on a variety of cancer cell lines [6]‐[9]. The key to the anti-tumour effect of ascorbic acid is the production of cytotoxic hydrogen peroxide [10,11]. Ascorbic acid has many known interactions with metal ions, catalysing its oxidation with concomitant formation of hydrogen peroxide, among other things. [12,13]. Chen et al. analysed the anticancer effect of extracellular ascorbic acid in pharmacological concentrations (up to 20 mmol/L), with the result that most cancer cells, but not normal cells, were affected by 20 mmol/L ascorbic acid, a concentration easily obtainable by intravenous injection [9].

In this paper we present a panel of 11 human cancer cell lines, carcinomas and glioblastomas, in which 55% of the cell lines were more susceptible (EC_50_ ≤ 20 mmol/L) and 45% were more resistant (EC_50_ >20 mmol/L) to the incubation with ascorbic acid. In addition, the two benign cell types (endothelial cells and fibroblasts) belong to the more resistant cell group. The reason for the resistance of some tumour cell lines and the benign cells to ascorbic acid mediated hydrogen peroxide production may be due to efficient antioxidant defences. Immunohistochemistry has shown that cancer cells can have elevated levels of antioxidant enzymes [14], but many of them seem to be deficient in catalase protein or catalase activity [15]. Therefore, the impact of intracellular catalase on preventing oxidative stress mediated by hydrogen peroxide must be analysed in more detail. We found that the 3 glioblastoma cell lines are extremely susceptible to ascorbic acid revealed reduced activity of intracellular catalase. In contrast, ascorbic acid resistant cancer cell lines, for example the breast carcinoma cell line BT-20, exhibited increased catalase protein and enzymatic activity. A catalase knockdown in BT-20 cells sensitized them to the toxic effect of extracellular ascorbic acid. The results indicate that catalase is important for the resistance of cancer cells to oxidative stress mediated by hydrogen peroxide.

## Material and methods

### Cell lines and reagents

**Figure 1:**
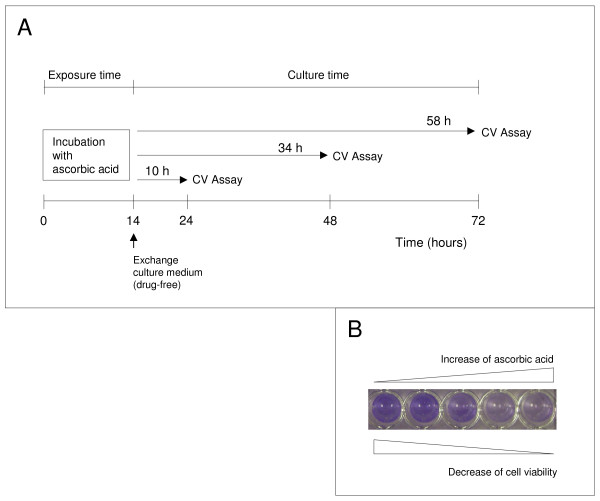
**The experimental design for measuring the ascorbic acid mediated cytotoxic effect. (A)** Cells in logarithmic growth phase were exposed to serial dilutions of ascorbic acid (5-100 mmol/L) for 14 h (exposure time). Afterwards, the cells were washed and cultured in cultured medium free of ascorbic acid, for 10, 34 and 58 h. **(B)** The number of viable cells was measured after culture by crystal violet (CV) staining with an ELISA reader.

Eleven malignant and 2 benign human cell lines were tested (Table 1). Cells were cultured at a cell density of 1.5 × 10^4^ cells per well of a 96-well plate at 37°C in 5% CO_2_ in their recommended growth media containing 10% FCS, 2 mmol/L glutamine (Invitrogen) and treated with ascorbic acid (5, 10, 15, 20, 25, 50, 100 mmol/L, pH 7; Sigma-Aldrich) for 14 h (Figure 1). Subsequently the medium was removed, the cells were washed once and cultured in growth medium without ascorbic acid for an additional 10, 34 and 58 h (Figure 1). Ascorbic acid was buffered to pH 7.0 with sodium hydroxide and prepared immediately before use. Selected cell lines (BT-20, SKOV-3, 23132/87, U-251, U-87) were also exposed to serial dilutions (10, 50, 100, 200 μmol/L) of hydrogen peroxide (Sigma-Aldrich) for 2 h. The medium was then removed and cells were washed and cultured in growth medium for an additional 22 h without hydrogen peroxide.

**Table 1:** Panel of human cell lines tested in this study

**Cell line**		**Cell line**	
23132/87	gastric carcinoma	HT-29	colon carcinoma
SKOV-3	ovarian carcinoma	BXPC-3	pancreas carcinoma
BT-20	breast carcinoma	MCF-7	breast carcinoma
U-13898	glioblastoma	MDA-MB-468	breast carcinoma
U-87	glioblastoma	MDA-MB-231	breast carcinoma
U-251	glioblastoma		
HUVEC	endothelial cells	NHDF	fibroblasts

The cells were purchased from different suppliers, e.g. American Type Culture Collection (http://www.atcc.org), Health Protection Agency Culture Collection (http://www.hpacultures.org.uk), German Collection of Microorganism and Cell Culture (http://www.dsmz.de) and PromoCell (http://www.promocell.com).

### Measurement of cytotoxicity

Effective concentration (EC_50_) values, which indicate the concentration of ascorbic acid that reduced the number of viable cells by 50%, were determined after culture (Figure 1) by the crystal violet assay [16]. This assay is based on the photometric measurement of crystal violet, which bonds at the DNA of viable cells. The measured OD values at a wave length of 570 nm are directly proportional to the number of viable cells. Data are presented as the mean ± standard deviation of hexaplicates for each ascorbic acid concentration. The experiments were repeated independently three times each.

### Determination of catalase levels

The level of catalase protein expression was detected by western blot analysis in the following cell lines: BT-20, SKOV-3, 23132/87, U-251, U-87. Cell pellets were lysed with the ready-to-use solution M-PER (Pierce, ThermoFisher Scientific). Protein concentration was determined by Bradford Assay (Pierce) and 20 μg of protein per slot was separated by SDS/Page and subsequently transferred onto nitrocellulose membrane (Whatman, GE Healthcare). Protein transfer was confirmed with the prestained protein ladder from Fermentas, Life Science (#SM0671). A polyclonal anti-human catalase antibody (diluted 1:200 (#sc-34282) Santa Cruz Biotechnology) and anti-human β-actin (diluted 1:200 (#sc-130301) Santa Cruz) were used as primary antibody, and a donkey anti-goat IgG secondary antibody coupled to horseradish peroxidase (1:20,000 (#sc-2020) Santa Cruz) was applied for one hour at room temperature. The enhanced chemiluminescent reagent ECL was used for detection (Amersham, GE Healthcare). Immunoblots were scanned and analysed by using Image J program provided by the National Institutes of Health. Relative expression level was determined by densitometry and normalized to the expression of β-actin.

### Inhibition of catalase gene expression by short hairpin RNA (sh-RNA)

Expression of catalase was knocked down with Q-tech by SIRION Biotech (http://www.sirion-biotech.de). Expression of catalase (NM_001752) was silenced in BT-20 cells by sh-RNA after transducing with adenoviral vector Ad-shCAT under the control of the human U6 promotor (performed by SIRION Biotech). BT-20 Ctrl cells were transduced with Q-tech control vector containing the non-target (NT) sh-RNA sequence CAACAAGATGAAGAGCACCAA. Virus production was carried out in HEK 293 cells.

### Catalase activity assay

Catalase activity was determined with a commercially available assay kit and was performed according the manufacture’s instructions (http://www.cellbiolabs.com). Cell Biolabs’ OxiSelect Catalase Activity Assay (#STA-341) involves two reactions. Cells were harvested with a rubber policeman and collected by centrifugation (2000 xg for 10 min at 4°C). The cell pellets were sonicated in 1 ml cold PBS and centrifuged at 10,000 xg for 15 min at 4°C. Twenty μl of supernatant were used for the assay. The first reaction is the catalase induced decomposition of known amounts of hydrogen peroxide into water and oxygen. The remaining hydrogen peroxide in the reaction mixture mediates a second reaction with a chromogenic reagent to a quinoneimine dye coupling product measuring at 520 nm. The rate of hydrogen peroxide disintegration is proportional to the concentration of catalase. Catalase activity was calculated with the following formula: B/30 × V × sample dilution factor = nmol/min/ml = mU/ml; B is the amount of decomposed hydrogen peroxide from hydrogen peroxide standard curve in mmol/L and V is the pretreated sample volume in ml added into the reaction; 30 is the reaction time, 30 min. Catalase activity was normalized for protein concentration (determined by Bradford Assay) and expressed as mU per 100 μg of protein.

### Determination of caspase activity

The Caspase-Glo 3/7 luminescent assay was performed according the manufacture’s instructions (http://www.promega.com). These members of the cysteine aspartic acid-specific protease (caspase) family play key effector roles in apoptosis in mammalian cells. The assay provides a proluminescent caspase-3/7 substrate, which contains the tetrapeptide sequence DEVD. This substrate is cleaved to release aminoluciferin, a substrate of luciferase used in the production of light. The generated luminescent signal is proportional to caspase-3/7 activity and was measured with a luminometer (Genios Pro; Tecan, Switzerland).

### Statistical analysis

GraphPad Prism 4.0 software (Statcon, Witzenhausen, Germany) was used for statistical analyses. Data were analysed by Mann-Whitney U test to show significant differences between the groups after the nonparametric rank variance test of Puri and Sen. Probability values below 0.05 were considered significant.

## Results

### The cytotoxic effect of ascorbic acid on different human cancer cell lines

**Figure 2:**
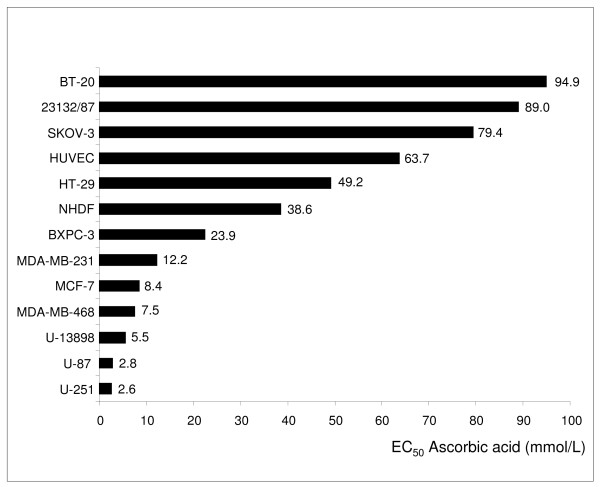
**Relative cytotoxicity of ascorbic acid on cancer and benigne cells.** Shown are the EC_50_ values of different cell lines for an ascorbic acid exposure time of 14 h and an ascorbic acid free culture time of 34 h. Cell viability was measured with the crystal violet assay at the end of culture. The results shown are representative for 3 independent analyses.

The toxic effect of ascorbic acid was analysed on 11 malignant and 2 benign cell lines (Table 1). For this purpose, the cells were exposed to ascorbic acid *in vitro* for 14 h, subsequently the medium was removed and the cells were cultured without ascorbic acid for an additional 10, 34 and 58 h (Figure 1) to determine the concentration that decreased cell survival to 50% (EC_50_). The tested cell lines demonstrated obvious differences in their resistance to ascorbic acid (Figure 2). Five cancer cell lines had an EC_50_ > 20 mmol/L (up to 20 mmol/L: the possible pharmacological concentration available by intravenous injection [9]) and within this group the 3 cell lines SKOV-3, 23123/87, and BT-20 demonstrated an EC_50_ > 79 mmol/L (Table 2). A moderate EC_50_ between 20 and 79 mmol/L was determined for the 2 cancer cell lines BXPC-3, and HT-29. Six cancer cell lines exhibited an EC_50_ < 20 mmol/L: U-251, U-87, U-13898, MDA-MB-468, MCF-7, and MDA-MB-231. The origin of the cancer cells did not explain their susceptibility to ascorbic acid mediated cytotoxicity. While the breast carcinoma cell line BT-20 was highly resistant to the exposure to ascorbic acid (EC_50_: 94.9 mmol/L), the breast carcinoma cell lines MDA-MB-231 (EC_50_: 12.2 mmol/L) and MDA-MB-468 (EC_50_: 7.5 mmol/L) were more susceptible. Fibroblasts and endothelial cells demonstrated EC_50_ values of 38.6 and 63.7 mmol/L, respectively (Figure 2).

**Table 2:** **Relative cytotoxicity of ascorbic acid and hydrogen peroxide (H**_**2**_**O**_**2**_**) on cancer cells**

**Cell line**	**EC**_**50**_**ascorbic acid (mmol/L)**	**EC**_**50**_**H**_**2**_**O**_**2**_**(μmol/L)**
BT-20	94.9	241,2
SKOV-3	79.4	172,0
23132/87	89.0	165,5
U-87	2.8	96.6
U-251	2.6	77.1

Five cancer cell lines, characterized by their different susceptibility to the ascorbic acid-mediated cytotoxic effect based on the generation of hydrogen peroxide, were analysed for their susceptibility to hydrogen peroxide-mediated cytotoxicity.

### Ascorbic acid resistant human cancer cell lines are cross-resistant to hydrogen peroxide

The toxicity of extracellular ascorbic acid is caused by the generation of hydrogen peroxide [10,11]. The ascorbic acid induced generation of extracellular hydrogen peroxide was successfully detected (not shown). Therefore, cancer cells lines resistant to the ascorbic acid mediated cytotoxic effect should also be more resistant to the toxic effect of hydrogen peroxide than ascorbic acid susceptible cell lines. To confirm this assumption, the 3 cancer cell lines BT-20, SKOV-3, 23132/89, more resistant to the toxic effect of ascorbic acid, and the 2 sensitive cell lines U-251, and U-87 were incubated with different concentrations of hydrogen peroxide. The cancer cell line BT-20, highly resistant to the toxic effect mediated by ascorbic acid, was also highly resistant to the toxic effect mediated by hydrogen peroxide (Table 2). In contrast, the glioblastoma cell lines U-251 and U-87, extremely susceptible to the ascorbic acid mediated cytotoxic effect (EC_50_ < 5.0 mmol/L), were most sensitive to hydrogen peroxide, too (Table 2).

**Figure 3:**
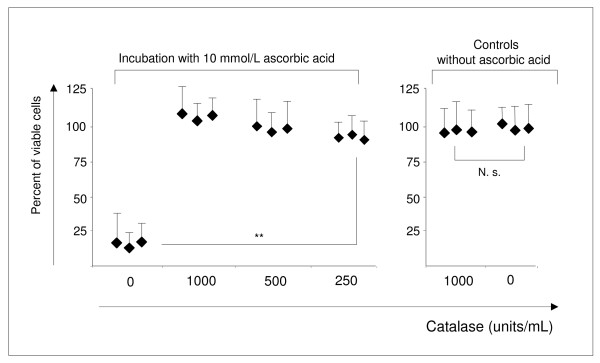
**The hydrogen peroxide scavenger catalase prevents the cytotoxic effect of ascorbic acid-mediated hydrogen peroxide production.** The addition of exogenous catalase to cells of the U-251 cell line incubated with toxic concentrations of ascorbic acid (10 mmol/L) prevented the lethal effect elicited by ascorbic acid. U-251 cells incubated with catalase alone were not affected in their viability. The results shown are representative for 3 independent analyses and values are expressed as mean ± standard deviation of hexaplicates. The difference between 0 and 250 units/mL catalase is significant (*p* = 0.004). N.s.: not significant.

Adding exogenous catalase to glioblastoma cell lines protected them against the toxic effect of ascorbic acid. The glioblastoma cell line U-251, extremely sensitive to the exposure to ascorbic acid (EC_50_: 2.6 mmol/L), was incubated with 10 mmol/L ascorbic acid, the toxic concentration for this cell line, and different concentrations of catalase (250 - 1000 U/mL) for 4 h. The cells were subsequently cultured for 20 h before measuring cell viability. The presence of exogenous catalase during exposure time (Figure 1) prevented the toxic effect of both ascorbic acid (Figure 3) and hydrogen peroxide (not shown). The same results were obtained for the cell line U-87 (not shown).

### Catalase protein and enzymatic activity in human cancer cells correlate with an increased resistance to ascorbic acid mediated cell toxicity

**Figure 4:**
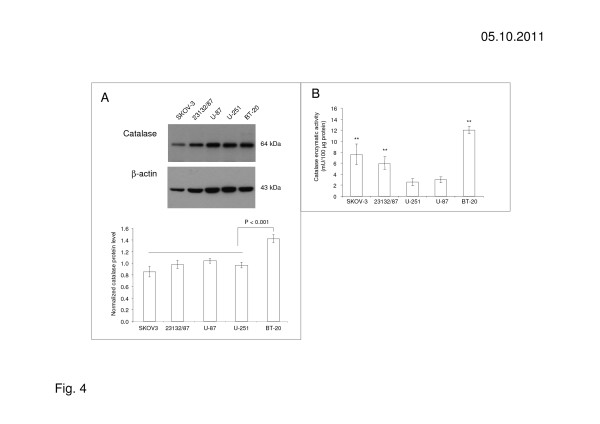
**Catalase protein and enzymatic activity in ascorbic acid resistant and susceptible cells. (A)** Immunoblots and densitometric analyses of catalase protein, and **(B)** enzymatic activity in ascorbic acid resistant cancer cell lines (BT-20, 23132/87, SKOV-3) and ascorbic acid susceptible cancer cell lines (U-251, U-87). The protein level of catalase in ascorbic acid resistant BT-20 cells is significantly different (*p* < 0.001) to the levels in 23132/87, SKOV-3 and the ascorbic acid susceptible cell lines U-251, U-87. There is a correlation between catalase activity and resistance to the ascorbic acid mediated cytotoxic effect. The results shown are representative for 3 independent analyses. Values are expressed as mean ± standard deviation and significant differences (p ≤ 0.01) are shown (**) compared to U-251 and U-87, respectively.

The addition of exogenous catalase to ascorbic acid susceptible cancer cell lines neutralizes the cytotoxic effect of ascorbic acid. Therefore, we investigated the assumption that ascorbic acid resistant cells protect themselves by increasing expression of intracellular catalase. For this, the 5 cell lines BT-20, SKOV-3, 23132/89, U-251, and U-87, characterized by different sensitivities to ascorbic acid, were examined concerning the catalase protein levels by immunoblot. Compared to other cancer cell lines, the level of catalase protein was significantly higher in BT-20 cells highly resistant to the ascorbic acid mediated cytotoxic effect compared to the other cancer cell lines (Figure 4). Since protein expression does not always correlate directly with enzymatic activity, an enzymatic assay was used to determine catalase activity. Catalase activity in the ascorbic acid resistant cell lines SKOV-3, 23132/87 and BT-20 was significantly increased in comparison to catalase activity measured in the ascorbic acid non-resistant cell lines U-87 and U-251 (Figure 4B).

### Silencing catalase expression in BT-20 cancer cells increased their susceptibility to the toxicity of ascorbic acid

**Figure 5:**
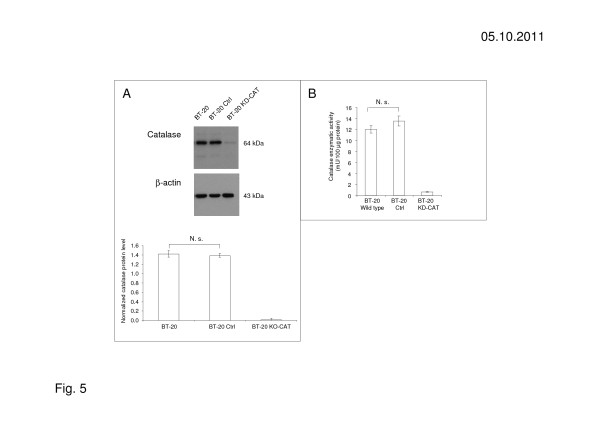
**Catalase-silencing influenced catalase expression and activity in BT-20 KD-CAT cells. (A)** Catalase knock-down by sh-RNA was proofed by western blot. **(B)** BT-20 KD-CAT cells demonstrated strongly reduced catalase activity in contrast to BT-20 wild type cells (BT-20 WT) and BT-20 control cells (BT-20 Ctrl) transduced with Q-tech control vector containing the non-target sh-RNA sequence CAACAAGATGAAGAGCACCAA. The results shown are representative for 3 independent analyses.

To study the possible protective role of catalase in ascorbic acid resistant cancer cell lines, the expression of catalase was silenced in the ascorbic acid resistant breast carcinoma cell line BT-20 with specific sh-RNA. BT-20 control cells (BT-20 Ctrl) was transduced with a control vector coding for non-target sh-RNA. The knock-down effect of sh-RNA transduction on the expression of catalase protein was proved by western blot (Figure 5). The maximum level of catalase knock-down was found to be 90% with RT-qPCR (not shown) and 95% in western blot (Figure 5A). In addition, the catalase enzymatic activity was reduced > 97% by knock-down (Figure 5B). BT-20 KD-CAT cells, BT-20 Ctrl cells and BT-20 wild type cells did not demonstrate obvious differences in cell morphology and cell growth (not shown).

**Figure 6:**
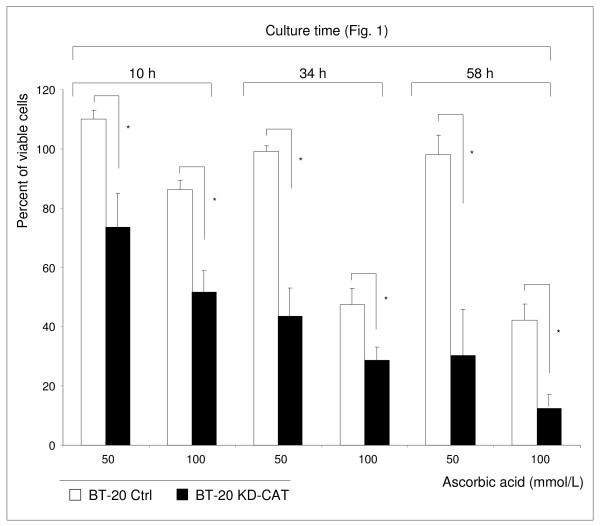
**Catalase-silenced BT-20 KD-CAT cells are sensitized to the toxic effect of ascorbic acid.** BT-20 KD-CAT cells were significantly more affected by the exposure to high concentrations of ascorbic acid (50 and 100 mmol/L) than BT-20 Ctrl cells. The results shown are representative for 3 independent analyses. Values are expressed as mean ± standard deviation. *p < 0.01.

**Figure 7:**
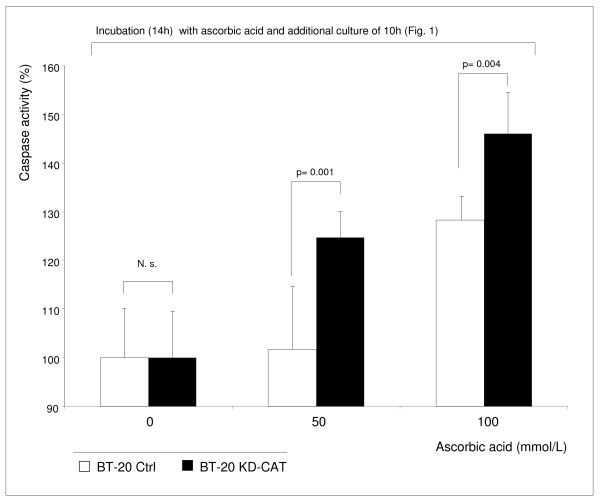
**Catalase-silenced BT-20 KD-CAT cells demonstrate increased caspase activity in the presence of ascorbic acid.** BT-20 KD-CAT cells and BT-20 Ctrl cells demonstrated significant differences in caspase activity in the presence of 50 mmol/L and 100 mmol/L ascorbic acid. At lower concentration (10 mmol/L), there were no significant differences (data not shown). The results shown are representative for 3 independent analyses. Values are expressed as mean ± standard deviation and significant differences are shown.

Catalase knock-down in BT-20 cells (BT-20 KD-CAT cells) was associated with increased susceptibility to the ascorbic acid mediated toxic effect (Figure 6). The BT-20 KD-CAT cells can be protected by external catalase against the toxic effect of both ascorbic acid and hydrogen peroxide acid (not shown). An ascorbic acid concentration of 50 mmol/L did not influence BT-20 Ctrl cells (Figure 6) and BT-20 wild type cells (Figure 2) but increased cell death in BT-20 KD-CAT cells (Figure 6). BT-20 cells are strongly resistant to ascorbic acid mediated oxidative stress (EC_50_: 94.9 mmol/L) and in the presence of 100 mmol/L ascorbic acid the cell viability of BT-20 KD-CAT cells decreased stronger than the viability of BT-20 Ctrl cells (Figure 6) and BT-20 wild type cells. However, a low percentage (< 20%) of BT-20 KD-CAT cells remained viable. We found that the enzyme activity of glutathione peroxidase, the second peroxide-detoxifying enzyme, was not influenced by catalase knock-down (Additional file 1: Figure S1). Therefore, we hypothesize that the remaining viability of BT-20 KD-CAT cells and BT-20 Ctrl cells was caused by the activity of glutathione peroxidase. Nevertheless, the data presented suggest that catalase plays an important role in the resistance to ascorbic acid mediated oxidative stress. In addition, the susceptible BT-20 KD-CAT cells demonstrated significantly higher caspase 3 and 7 activity in the presence of 50 and 100 mmol/L ascorbic acid in comparison to BT-20 Ctrl cells (Figure 7). These findings demonstrate that inhibition of catalase in strongly resistant BT-20 cells (BT-20 KD-CAT cells) sensitizes them to ascorbic acid mediated oxidative stress and increases the rate of apoptosis.

## Discussion

The key to the anti-tumour effect of ascorbic acid is the production of cytotoxic hydrogen peroxide [10,11]. In this study a panel of 11 human cancer cell lines was tested for their susceptibility to ascorbic acid. Three glioblastoma cell lines and the 3 breast carcinoma cell lines demonstrated EC_50_ values < 20 mmol/L and were obviously susceptible to ascorbic acid mediated cytoxtoxicity. Five of 11 carcinoma cells lines with EC_50_ values > 20 mmol/L were only marginally influenced in their viability by elevated ascorbic acid concentrations (Figure 2).

In accordance with previous studies [9]‐[11,13], we found a toxic effect of ascorbic acid based on the local production of hydrogen peroxide. Cell lines, e.g. BT-20, 23132/87, SKOV-3, with a natural resistance to the incubation with ascorbic acid, also demonstrated a natural resistance to toxic effects mediated by hydrogen peroxide (Table 2). In contrast, cell lines, e.g. U-251 and U-87, susceptible to the incubation with ascorbic acid, were also more susceptible to the incubation with hydrogen peroxide. In addition, ascorbic acid resistant cancer cell lines are more able to protect themselves with increased catalase enzymatic activity, in contrast to ascorbic acid susceptible cancer cell lines (Figure 4B). Catalase-silencing sensitizes BT-20 breast carcinoma cells to ascorbic acid mediated cell death. In addition to catalase, enzymes of the peroxidase family, e.g. glutathione peroxidase, are also important for cell protection. In the present study, expression of glutathione peroxidase was also proofed for all tested cancer cell lines, but the level of protein and enzymatic activity did not strongly correlate with the resistance of cancer cell lines to the ascorbic acid-mediated cytotoxic effect (not shown). The catalase knock-down in BT-20-KD-CAT cells did not influence glutathione peroxidase activity (Additional file 1: Figure S1), suggesting that glutathione peroxidase may not play a major role in protecting cancer cells against cytotoxic hydrogen peroxide.

Ascorbic acid is able to act as a strong electron donator by reducing iron ions (Fe^3+^ to Fe^2+^). These ions may exist alone or bound on matrix metal proteins [12]. Other metal ions like Cu^2+^, Ti^3+^, Cr^2+^ or Co^2+^ can also be used as an electron carrier. These ions can be oxidized and donate their electrons on oxygen by generating a superoxide anion (O^2-^). Superoxide dismutase catalyses the reaction of O^2-^ to hydrogen peroxide that can induce apoptosis in different ways: blocking the activity of a plasma membrane Na^+^/H^+^ exchange system leading to reduced cytosolic pH values or attacking DNA, usually by its conversion into DNA-damaging hydroxyl ion (OH^·^) [17]. In the present study we found that extracellular catalase prevented the cell toxic effect of ascorbic acid and supported cell viability of ascorbic acid susceptible cancer cell lines (Figure 3). Catalase catabolizes hydrogen peroxide to water and oxygen and helps to protect aerobic organisms against excessive hydrogen peroxide production. The cytotoxic effect of extracellular ascorbic acid is finally mediated by the development of extracellular hydrogen peroxide which is membrane permeable [18]. In addition, it is well known that ascorbic acid enters directly into the cell with sodium-dependent vitamin C transporter (SVCT1 and SVCT2) and in its oxidized form dehydro-ascorbic acid can be internalized by hexose transporters GLUT 1, GLUT 3, and GLUT 4 [19]. Both ascorbic acid and its oxidized form are in extra- and intracellular balance, depending on their pH-value. The extracellular amount of ascorbic acid was identified as the more important one, because ascorbic acid has toxic effects on cells even if there is only little expression of those transporters [9,20].

It seems that many cancers demonstrate substantially lower catalase activity than normal tissues, allowing cancers to generate a moderate intracellular level of oxidative stress to aid their proliferation and survival [15,21]. It is known that expression of catalase is regulated at message, protein and activity levels [22]. We could show that the tumour cell lines used in the present study are different in their catalase activity. Szatrowski described that rapidly proliferating cells such as cancer cells generate abnormally high hydrogen peroxide levels. This and other factors increased oxidative stress during neoplastic transformation and may promote the selection of cells with modified (increased or decreased) catalase activity. The modified catalase expression in cancer cells remains puzzling but it seems that prolonged exposure to reactive oxygen species (ROS) downregulates catalase expression via hypermethylation of the catalase promoter and, in addition, transcription factors seem to be involved [23,24]. Catalase is also down-regulated in healthy cells transformed with T-antigen of SV40 or Ras, although the underlying mechanisms of this down-regulation are still unknown [25]. Interestingly, it also has been observed that catalase levels are modified in cancer cell lines resistant to some chemotherapeutic agents or hydrogen peroxide [26,27]. In summary, catalase expression is regulated in a wide array of cellular processes.

The use of ascorbic acid in tumour therapy is a matter of some controversy [28]‐[31]. Nevertheless, ascorbic acid is used in tumour therapy, especially when evidence based medicine or supportive therapy fail [32,33]. Many conventional and novel anti-cancer drugs have been reevaluated for their association with ROS production. For instance, doxorubicin is a redoxcycling anthracycline that generates ROS. Biologics can also induce apoptosis through the generation of ROS. Rituximab, an anti-CD20 monoclonal antibody approved for the treatment of non-Hodgkin’s lymphoma, induces a rapid and intense production of ROS in human lymphoma cells [34]. Another aspect of ROS is that they are able to provoke uncontrolled cell growth by overstimulation of MAP Kinases signal transduction pathways [35]‐[38]. Furthermore, ROS can activate hypoxia induced factor 1 (HIF-1) that stimulates the cells to gain energy from glucose under hypoxic conditions. HIF-1 increases the expression of glycolysis enzymes and additionally stimulates the development of new blood vessels (neovascularisation) by increasing the expression of angiogenic factors (e.g. VEGF) to enhance oxygen supply [39,40]. Increased levels of ROS, however, damage cell structure and function [40].

On the basis of our data, we were able to show a correlation between catalase activity and resistance of cancer cell lines to the ascorbic acid induced cytotoxic effect. Moreover, catalase is significant for cell protection against hydrogen peroxide. The ascorbic acid resistant cell line BT-20 became more susceptible to ascorbic acid after sh-RNA mediated catalase knock-down and the rate of apoptosis increased in these cells.

## Conclusions

The present study demonstrates great differences in the ability of cancer cell lines to prevent cell damage induced by increased levels of hydrogen peroxide induced by ascorbic acid. Forty-five percent of the cancer cell lines tested are not affected by ascorbic acid and hydrogen peroxide, respectively. Higher levels of catalase activity are found in cell lines that are more resistant to oxidative stress than in more susceptible cancer cell lines. This observation underlines the heterogeneity of cancer cells concerning their ability to prevent cell death induced by oxidative stress. Therefore, anticancer therapies based on increased generation of ROS are influenced in their efficacy by the antioxidative defence potential of cancer cells. In this context the results of the present study underline the important function of catalase as an antioxidative enzyme.

## Competing interests

The authors declare no conflict of interest.

## Authors’ contributions

CK and CO drafted the manuscript, designed the study, set up the experiments, participated in data collection, analysed and interpreted the results and provided images and figures. MK, UK, BM, MS, and MiK carried out experiments and participated in data interpretation. UK, ACK and CTG revised the article for intellectual content and participated in editorial support. All authors read and approved the final manuscript.

## Pre-publication history

The pre-publication history for this paper can be accessed here:

http://www.biomedcentral.com/1472-6882/12/61/prepub

## Supplementary Material

Additional file 1 **Figure S1. Glutathione peroxidase activity in BT-20 KD-CAT cells, BT-20 control cells and BT-20 wild type cells.** The knock-down of catalase does not influence glutathione peroxidase activity, suggesting that glutathione peroxide may not play a major role in resistance to oxidative stress. Glutathione peroxidase was measured with BioVision’s Glutathione Peroxidase Activity Assay (#K762-100) according the manufacture’s instructions (www.biovision.com). For this, one million cells were homogenized in 200 μl cold assay buffer on ice, centrifuged at 10,000 xg for 15 min at 4°C and 50 μl of the supernatant were used for the assay. Glutathione peroxidase reduces hydrogen peroxide while oxidizing reduced glutathione (GSH) to oxidized glutathione (GSSG). The generated GSSG is reduced to GSH with consumption of NADPH by glutathione reductase. The decrease of NADPH, measured at 340 nm, is proportional to glutathione peroxidase activity. Glutathione peroxidase activity was normalized for protein concentration (determined by Bradford Assay) and expressed as mU per 100 μg of protein. The results shown are representative for 3 independent analyses. Click here for file
