# Supplementary figures and images for: Natural resistance to ascorbic acid induced oxidative stress is mainly mediated by catalase activity in human cancer cells and catalase-silencing sensitizes to oxidative stress
Source: BMC Complement Altern Med. 2012 May 2;12:61. doi: 10.1186/1472-6882-12-61 (PMC3404974; doi:10.1186/1472-6882-12-61)

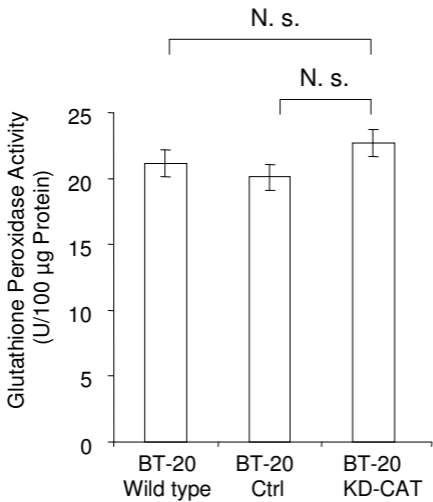

Supplement: Additional file 1 — Figure S1. Glutathione peroxidase activity in BT-20 KD-CAT cells, BT-20 control cells and BT-20 wild type cells. The knock-down of catalase does not influence glutathione peroxidase activity, suggesting that glutathione peroxide may not play a major role in resistance to oxidative stress. Glutathione peroxidase was measured with BioVision’s Glutathione Peroxidase Activity Assay (#K762-100) according the manufacture’s instructions (www.biovision.com). For this, one million cells were homogenized in 200 μl cold assay buffer on ice, centrifuged at 10,000 xg for 15 min at 4°C and 50 μl of the supernatant were used for the assay. Glutathione peroxidase reduces hydrogen peroxide while oxidizing reduced glutathione (GSH) to oxidized glutathione (GSSG). The generated GSSG is reduced to GSH with consumption of NADPH by glutathione reductase. The decrease of NADPH, measured at 340 nm, is proportional to glutathione peroxidase activity. Glutathione peroxidase activity was normalized for protein concentration (determined by Bradford Assay) and expressed as mU per 100 μg of protein. The results shown are representative for 3 independent analyses. [file 1472-6882-12-61-S1.pdf]
